# Telerehabilitation for Total Hip and Knee Arthroplasty Patients: A Pilot Series with High Patient Satisfaction

**DOI:** 10.1007/s11420-019-09715-w

**Published:** 2019-08-21

**Authors:** Justin Kuether, Anne Moore, Joseph Kahan, Joseph Martucci, Tara Messina, Roland Perreault, Robert Sembler, John Tarutis, Bohdanna Zazulak, Lee E. Rubin, Mary I. O’Connor

**Affiliations:** Center for Musculoskeletal Care, Department of Orthopaedics and Rehabilitation, Yale School of Medicine and Yale New Haven Health, New Haven, CT USA

**Keywords:** total joint arthroplasty, telerehabilitation

## Abstract

**Background:**

The demand for total hip and total knee arthroplasty in the USA is projected to increase significantly. Traditionally, face-to-face physical therapy has been an essential component of recovery in patients after total joint arthroplasty. Emerging technology allows telerehabilitation, or virtual physical therapy, which may reduce costs and increase standardization, but its effects on outcomes are not known.

**Questions/Purpose:**

We sought to review our initial experience using a telerehabilitation protocol for patients after primary total hip or total knee arthroplasty.

**Methods:**

In this pilot study, we retrospectively compared our first 40 telerehabilitation patients after a primary total hip or knee arthroplasty with a historical cohort or literature referenced values and evaluated (1) readmission rates at 90 days, (2) emergency department visits, (3) patient-reported outcome scores, (4) incidence of closed knee manipulation within 90 days of primary total knee arthroplasty, and (5) patient satisfaction surveys.

**Results:**

We observed no increase in the telerehabilitation group at 90 days in readmissions, emergency department visits, or closed knee manipulations. Accuracy of telerehabilitation exercises performed was 92%. Patient-reported outcome scores showed improvements comparable with traditional therapy. Extremely high patient satisfaction scores were reported with the telerehabilitation protocol.

**Conclusion:**

Our early experience demonstrates the feasibility of implementing a telerehabilitation program following primary total hip or knee arthroplasty without compromising clinical quality and with high patient satisfaction.

**Electronic supplementary material:**

The online version of this article (10.1007/s11420-019-09715-w) contains supplementary material, which is available to authorized users.

## Introduction

The demand for total hip and total knee arthroplasty in the USA is projected to increase significantly in the coming years [5]. Correspondingly, this growth will include a greater number of outpatient total joint arthroplasty procedures [1]. Even with a shift to same-day surgeries, the projected volume increase in hip and knee arthroplasty will create financial challenges to both government and private insurers. Patients may also incur higher out-of-pocket expenses, given the growth of high-deductible health plans with co-payments for physical therapy sessions. Furthermore, some hospitals are at financial risk due to mandated participation in bundled payment models for lower extremity arthroplasty. Thus, the development of methods to deliver high-quality care at reduced costs is essential for multiple stakeholders.

Traditionally, face-to-face physical therapy, whether home-based or outpatient, has been the standard of care to promote recovery after these surgeries [3, 6, 11]. Such sessions between the patient and the physical therapist constitute a portion of post-discharge costs; post-discharge costs make up more than 35% of the disbursements for total joint arthroplasty procedures [2]. Furthermore, there has been little standardization in the duration of therapy or the exercises that constitute it [6, 16]. The use of telerehabilitation may promote standardization, improve patient compliance with exercise regimens, and lower costs. Additionally, such technology may also facilitate participation in post-operative rehabilitation when patients cannot access traditional physical therapy services due to geographic challenges or inability to travel [13].

Telerehabilitation is well established as a viable strategy in total joint arthroplasty. Several well-designed studies demonstrate that in-home telerehabilitation is at least as effective as traditional face-to-face physical therapy following primary total joint arthroplasty [9, 10, 12, 14]. Additionally, patients have reported being satisfied with the experience of using telerehabilitation [15].

At our institution, we began a telerehabilitation pilot program in primary total hip and knee arthroplasty using a remotely managed in-home technology system (Reflexion Health, San Diego, CA, USA). The system provides a patient with at-home physical rehabilitative therapy via an animated avatar—a virtual therapist. The system, called VERA™, which stands for Virtual Exercise Rehabilitation Assistant, allows a patient to participate in a specific therapist-assigned routine, and the results are overseen by a live physical therapist. Face-to-face contact can be initiated by either the physical therapist or the patient. The telerehabilitation kit contains a screen that allows the patient to view the exercises, and built-in cameras record and track the patient’s movements. The units are self-contained and can be used even if the patient does not have in-home Wi-Fi service. In each session, the patient follows the avatar through the exercises, and the technology tracks the patient’s exercises and allows for feedback on the quality of many of the exercises performed.

We sought to retrospectively evaluate our experience using telerehabilitation with the VERA system in patients following primary total hip and knee arthroplasty. We hypothesized that in our initial cohort of 40 patients, telerehabilitation would deliver equivalent high-quality care at reduced cost. To evaluate this hypothesis, we compared this group to a retrospective historical cohort and evaluated (1) readmission at 90 days after a primary total hip or total knee arthroplasty; (2) validated patient-reported outcomes as measured by the abbreviated Hip Disability and Osteoarthritis Outcome Score and Knee Injury and Osteoarthritis Outcome Score for joint replacement (HOOS JR and KOOS JR, respectively) [7, 8, 13, 14]; (3) incidence of closed knee manipulation during 90 days after a primary total knee arthroplasty; and (4) patient satisfaction scores.

## Materials and Methods

The study was conducted at a single center and designed as a pilot study. Patients scheduled for unilateral primary total hip or total knee arthroplasty who were able to ambulate without an assistive device at baseline, acknowledged having caregiver support at home, and lacked any significant cardiovascular or pulmonary co-morbidities were offered enrollment in our telerehabilitation pilot. Forty patients who participated over 90 days from the time of surgery were recruited to the telerehabilitation group (TR group). These results were then retrospectively compared with those of 614 patients from the same year who were discharged home from the hospital and underwent traditional home and/or outpatient physical therapy (STD group).

Patients in the TR group had a mean age of 62.4 years and included 22 women and 18 men; those in the STD group had a mean age of 64.8 years and included 370 women and 244 men (Table 1). Acute hospitalization outcomes were similar between the groups (Table 2).

**Table 1:** Patient demographics

	VERA TJA patients	Traditional therapy TJA patients, home discharge
Volume	40	614
TKA	24	430
THA	16	184
Average age	62.4 years	64.8 years
Female	22 (55.0%)	370 (60.3%)
Male	18 (45.0%)	244 (39.7%)
Race		
White	35 (87.5%)	496 (80.8%)
Black	3 (7.5%)	82 (13.3%)
Asian	NA	6 (1.0%)
Other	NA	22 (3.6%)
Refused	2 (5.0%)	8 (1.3%)

*VERA* virtual exercise rehabilitation assistant, *TJA* total joint arthroplasty, *TKA* total knee arthroplasty, *THA* total hip arthroplasty, *NA* not available

**Table 2:** Acute care outcomes compared between telerehabilitation patients and traditional therapy patients

Metric	Telerehabilitation patients	Traditional therapy patients discharged to home	*p* value
Volume	40	614	
	*N* (%)	*N* (%)	
Unexpected return to OR	0	1 (0.2%)	1
Unplanned return to ICU	0	1 (0.2%)	1
Transfusion rate	1 (2.5%)	6 (1.0%)	0.358
Pneumonia	0	0	
Pulmonary embolism, deep venous thrombosis	0	0	
Sepsis	0	0	
Hematoma	0	0	
Surgical site infection	0	0	
Myocardial infarction	0	0	
Mechanical complication	0	2 (0.3%)	1
Length of stay	2.2	2.2	
Readmission 30 days	1 (2.5%)	26 (4.2%)	0.498
Mortality acute care 30 days	0	1 (0.2%)	0.939
Readmission 90 days	1 (2.5%)	35 (5.7%)	0.337
ED visit within 90 days (includes readmissions)	4 (10%)	60 (9.8%)	1

*OR* operating room, *ICU* intensive care unit

The protocol for the TR group included daily exercises with VERA, the telerehabilitation avatar, as well as a limited number of face-to-face post-operative physical therapy sessions. Telerehabilitation was considered complete when the patient had gone through the protocol and both therapist and patient felt comfortable with the patient’s progress (average duration, 8 weeks). The system recorded compliance, accuracy, and functional outcomes throughout usage. HOOS JR and KOOS JR surveys were completed pre-operatively, immediately post-operatively, and at the end of therapy. Patient satisfaction surveys were completed at the end of therapy when the telerehabilitation equipment was collected. Results were compared against literature norms.

Patient enrollment was voluntary. A patient who elected to participate in the telerehabilitation pilot met pre-operatively with the outpatient physical therapy coordinator, who performed the initial physical therapy assessment and assigned the telerehabilitation exercise program. The patient then had the telerehabilitation kit brought to their home by a Reflexion technician.

Surgeries were performed by a number of fellowship-trained arthroplasty surgeons. While in the hospital following surgery, all patients received the same in-house physical therapy; however, patients in the TR group were discharged with a specific physical therapy plan utilizing VERA, which the physical therapist could alter as the patient progressed. The assigned physical therapist could monitor recorded results of the patient’s activity during the course of treatment. Communication could be initiated through the VERA system or through traditional means (telephone, EPIC MyChart). To ensure clinical progress and both patient and surgeon comfort with the pilot program, the protocol also included a limited number of face-to-face physical therapy sessions over 8 weeks (maximum of six sessions for total hip arthroplasty and eight for total knee arthroplasty) to complement the telerehabilitation program. The STD group received approximately 16 visits for total knee arthroplasty and 12 visits for total hip arthroplasty over the same period. Outcome scores on KOOS JR and HOOS JR were collected pre-operatively, 2 weeks following surgery, and at completion of telerehabilitation. Patients completed a satisfaction survey shortly prior to the monitor being removed from their home by a technician.

Fisher exact tests were performed for comparison between the groups. All statistical analyses were performed using Stata 13.1 (StataCorp, College Station, TX, USA). Statistical significance was set as *p* < 0.05, two sided.

## Results

No differences were seen in acute care outcomes between the TR and STD groups (Table 2). In the TR group, there was one (2.5%) readmission at 30 days and one (2.5%) within 90 days, compared with 26 (4.2%) readmissions at 30 days and 35 (5.7%) readmissions at 90 days in the STD group. These differences were not statistically significant. The TR group had four (10%) ED visits compared with 26 (9.8%) in the STD group, which also was not statistically significant. None of the total knee arthroplasty patients required a post-operative manipulation.

The data collected from the telerehabilitation kits showed a wide range of adherence to the program (range, 12 to 92%; Table 3). However, it should be noted this range is not an accurate reflection of the use of the program by patients as it failed to account for time spent utilizing traditional therapy. Accuracy of the exercises performed with VERA was recorded at a mean of 92%, suggesting that participants were able to perform the exercises well at home using the computer as their guide.

**Table 3:** Metrics recorded by the telerehabilitation system

Process metrics	Mean	Range	Median
Episodic length	61 days	16–104	60
Episodic adherence	61%	12–92%	61%
Episodic accuracy	92%	78–98%	94%
Total logins	60.5	9–110	56.5
Total exercise hours	17.1	1.5–80.7	13.9
Daily minutes	21	13–55	17.3

In the TR group, HOOS JR and KOOS JR scores showed improvements comparable with those typically seen with traditional therapy (Table 4) [4]. Patient willingness to recommend the telerehabilitation program was evaluated using the Net Promoter Score and received an extremely favorable 90.3 (a score higher than 70 is considered “world class”).

**Table 4:** Average patient-reported outcomes at the completion of telerehabilitation

THA *N*=16	Initial HOOS JR	Last HOOS JR before VERA discharge	
Virtual therapy system scores	14.6	4.4	Min/max range HOOS JR 0–24
TKA *N*=22*	Initial KOOS JR	Last KOOS JR before VERA discharge	
Virtual therapy system scores	13.7	4.6	Min/max range KOOS JR 0–28

*KOOS JR* Knee Injury and Osteoarthritis Outcome Score, *HOOS JR* Hip Disability and Osteoarthritis Outcome Score, *TKA* total knee arthroplasty, *THA* total hip arthroplasty

## Discussion

A growing body of research supports the effectiveness of in-home telerehabilitation to reduce the cost and burden of post-operative rehabilitation in orthopedic procedures, specifically total hip and knee arthroplasty. Several studies have demonstrated its non-inferiority when compared with traditional face-to-face therapy on a variety of metrics [9, 10, 12, 14]. Our pilot study adds to this body of knowledge.

There are several limitations of this study. The pilot was neither a randomized controlled trial nor a matched cohort study; it was a pilot study with relatively small numbers into which selection bias may have been introduced. Additionally, there were some limited face-to-face sessions to complement the telerehabilitation protocol. On days when traditional therapy was conducted, the virtual therapy would have recorded a non-usage day, clouding our evaluationof true adherence to the program. Additionally, we could not discount the possibility that the satisfactory clinical outcomes seen in the TR group were due to traditional physical therapy sessions and not the study protocol focused on telerehabilitation.

Despite these limitations, in the TR group, we found no increase in readmissions, emergency department visits, or post-operative knee manipulations during the first 90 days following surgery and patient-reported outcomes consistent with those found using traditional physical therapy [4]. Additionally, patients reported extremely high satisfaction with the telerehabilitation experience.

We structured our pilot to recognize concerns that surgeons may have regarding decreased face-to-face physical therapy sessions. Our protocol included six to eight traditional face-to-face outpatient physical therapy visits over 8 weeks post-operatively. As we have gained experience, we have modified our telerehabilitation protocol to decrease the number of traditional therapy sessions to two to four sessions after total joint arthroplasty. Additionally, in the future, we will seek to incorporate pre-operative rehabilitation as part of the protocol in an effort to maximize post-operative results.

In conclusion, our pilot study of telerehabilitation after primary total hip and knee arthroplasty had very favorable outcomes. Patients like the convenience of the telerehabilitation program, and surgeons and physical therapists are comfortable with the protocol and confident that it can achieve excellent outcomes. Further study will be required to determine the optimal blend of traditional face-to-face, virtual, and telerehabilitation physical therapy sessions, as well as to conduct an in-depth cost analysis to evaluate the financial implications of adopting a telerehabilitation program.

## Electronic supplementary material

ESM 1 (PDF 1224 kb)

ESM 2 (PDF 1224 kb)

ESM 3 (PDF 1224 kb)

ESM 4 (PDF 1224 kb)

ESM 5 (PDF 1224 kb)

ESM 6 (PDF 1224 kb)

ESM 7 (PDF 1224 kb)

ESM 8 (PDF 1224 kb)

ESM 9 (PDF 1224 kb)

ESM 10 (PDF 1224 kb)

ESM 11 (PDF 1224 kb)
